# Driving factors on accumulation of cadmium, lead, copper, zinc in agricultural soil and products of the North China Plain

**DOI:** 10.1038/s41598-023-34688-6

**Published:** 2023-05-08

**Authors:** Zheng Liu, Ying Bai, Junhong Gao, Jun Li

**Affiliations:** 1 School of Chemical Engineering, Lanzhou City University, Lanzhou, 730070 China; 2 Research Center for Environmental Pollution Control of Yellow River Basin Cities, Lanzhou City University, Lanzhou, 730070 China; 3 Gansu Academy of Eco-Environmental Sciences, Chengguan District, Lanzhou, 730000 China; 4 Baiyin Ecological Environment Monitoring Center of Gansu Province, Baiyin, 730900 China

**Keywords:** Ecology, Environmental sciences

## Abstract

The accumulation of heavy metals in agricultural soils concerns food security. By using the Geographical Detector, this study investigated the influence of six types of factors (eleven factors) on the accumulation of Cd, Pb, Cu, Zn in agricultural soil and products of the North China Plain and confirmed the dominant factor. The results showed that heavy metals had accumulated in regional agricultural soils and the accumulation of Cd was severe. The accumulation of heavy metals was significantly influenced by policy factors (the management and reduction in usage of fertilizers and pesticides), fertilization factors (application of organic and chemical fertilizers), pesticide factors (application of herbicide and insecticide) and atmospheric deposition factors (heavy metal concentration in atmospheric deposition). The policy factor dominated the other three types of factors. Atmospheric deposition and the excess application of fertilizers and pesticides directly lead to the accumulation of heavy metals. Due to the high concentrations of heavy metals and abundant application amounts, organic fertilizers have contributed high levels of heavy metals to agricultural soils. This study suggests that formulated fertilization and action plans for pesticide reduction could effectively decrease the accumulation of heavy metals in agricultural soils and products in the study area.

## Introduction

Involving the safety of agricultural products, heavy metal pollution in agricultural soils has received much attention in China. A communique based on the first national survey of soil pollution status from 2005 to 2013 was issued by the Ministry of Environmental Protection and Ministry of Land and Resources of China^1^. The communique showed that 13.3% of national soil was contaminated by heavy metals, and the typical heavy metals in contaminated arable land were Cd, Ni, Cu, As, Hg and Pb. The study^2^ conducted a review of researches on farmland soil pollution in China from 2000 to 2018, revealing that the mean concentration of Cd in farmland was 0.86 mg kg^−1^. The mean value surpassed the risk screening threshold (0.6 mg kg^−1^) for soil contamination in Chinese agricultural land^3^. A separate study^4^ indicated that the farmland located within the city center perimeter in the northern region of the North China Plain served as a primary zone for heavy metal enrichment. Heavy metals could inhibit crop growth^5^, thereby reducing crop yields. Besides, heavy metals in agricultural products would accumulate in human body, inducing toxic effects. Exceedance of heavy metal limits in food crops was more common in southern China than in other regions^6^. This was partly because of high phytoavailability of heavy metals caused by soil acidification^7^. China has only 8.2% of the world’s arable land but has approximately 18.1% of the world’s population. Heavy metal pollution in arable land is an important issue for the survival of Chinese people.

Heavy metals in agricultural soils come from two types of sources: natural and anthropogenic. Natural sources are associated with soil types and soil parent material^8^. For example, Cr and Ni in soil were mainly affected by parent materials of Weifang, China^9^, and Cu and Zn were of geogenic origin in agricultural soils of Sialkot, Pakistan^10^. Anthropogenic sources, rather than natural sources, contribute most heavy metals to agricultural soils. Metal smelting highly affected Cu, Pb, Zn and As in the agricultural soil of the Shangdan Valley, Northwest China^11^. Vehicle exhaust is one of the main anthropogenic sources of heavy metals in agricultural highway soils in Jordan^12^. Atmospheric deposition was the dominant element source of heavy metals, including Cd, Hg, As, Cu, Pb, Zn, Cr and Ni, in agricultural soils in Heilongjiang and Zhejiang, China^13,14^. Agricultural production activities also had great effects on heavy metal input to agricultural soils. The accumulation of heavy metals in the facility agriculture soil of Shouguang, China, was related to the application of organic fertilizer, phosphate fertilizer and compound fertilizer^15^. Pesticides were suggested as one of the important anthropogenic sources of heavy metals in agricultural soils^16^. Straw return was demonstrated to lead directly to notable Cd accumulation in agricultural soils of the Jianghan Plain in central China^17^. Irrigation water was the main source of heavy metals (As, Cd, Cu and Hg), contributing 60–71% of the total inputs to agricultural soils in the Yangtze River delta, China^18^. In addition, the application of biosolids, sewage irrigation and waste disposal also influenced the accumulation of heavy metals in agricultural soils^8^.

The main sources of heavy metal were different from agricultural soils in different regions. Atmospheric deposition was the most important source of heavy metals in agricultural soils of China, but organic and chemical fertilizer and pesticides were the predominant sources in European countries^19^. For Cd, the main sources were atmospheric deposition, irrigation and livestock manure application in China and atmospheric deposition, chemical fertilizer application and irrigation in Europe^20^. In North China, atmospheric deposition contributed most heavy metals to agricultural soils due to highly developed heavy industry and more coal combustion, and in South China, the contribution of livestock manures was obviously higher because of flourishing agricultural production and animal husbandry^21^. The contribution rates of road dust and solid waste were higher for Pb than for other heavy metals in peri-urban agricultural soils under the great influence of human activities^22^. In addition, the main sources of different heavy metals were also different. Atmospheric deposition contributed the most proportions of Cd, Cr and Hg to paddy fields, but the secondary sources were irrigation water for Cd and fertilizer for Cr and Hg^23^.

Stopping the sources of heavy metals was suggested as the primary strategy to control pollution in agricultural soils^6^. Some typical policies conducted by the Chinese government were conducive to reducing the sources of heavy metals. In 2013, the Action Plan of Air Pollution Prevention and Control was published and implemented^24^. The Action Plan focused on reducing atmospheric particulates through the reduction of coal use and control of dust. In 2017, the aim of the Action Plan was completely achieved. The atmospheric deposition in nationwide agricultural soils was also effectively reduced with implementation of the Action Plan. In China, reducing the use of fertilizer is conducted mainly through soil testing and formulated fertilization (STFF). The STFF has been deployed around the country since 2005^25^. The STFF included measuring soil nutrients, developing a fertilization schedule and applying fertilizer in the field. The application of STFF could increase soil fertility and crop yield and quality through rational application of fertilizer. The application amount of fertilizer decreased through the application of STFF. Since 2015, in addition to STFF, a reasonable increase in the application of organic fertilizer and straw was commonly suggested to substitute the application of chemical fertilizer to reduce the application amounts of chemical fertilizer. Since the same year, a series of measures have been carried out to reduce the use of pesticides in agricultural production^26^. The action plan of pesticide reduction (APPR) until 2025 was also announced by the Ministry of Agricultural and Rural Affairs of China in 2022^27^. The measures included suiting the remedy to the case, accurately forecasting plant diseases and insect pests, cultivating disease-resistant and insect-resistant varieties, using efficient spraying devices of pesticides, promoting green prevention and control technologies, etc. These measures were commonly conducted with the application of STFF.

To date, many studies have been conducted on the source apportionment of heavy metals in agricultural soils. However, the sources were different among regions due to unbalanced regional development of agriculture, industry and society. Furthermore, the policy executive strength of the local government largely affected the source reduction of heavy metals in agricultural soils. Therefore, it was essential to conduct a study in a representative region. In this study, a traditional agricultural region in the North China Plain was selected as the study area. The driving factors of the accumulation of Cd, Pb, Cu and Zn in agricultural soil and products were confirmed by using the Geographical Detector. The interaction and difference between the driving factors were also investigated to determine the dominant factor. The results of this study provide a scientific basis for the management of heavy metal sources to prevent heavy metal accumulation in regional agricultural soil and products.

## Materials and methods

### Study area

**Figure 1:**
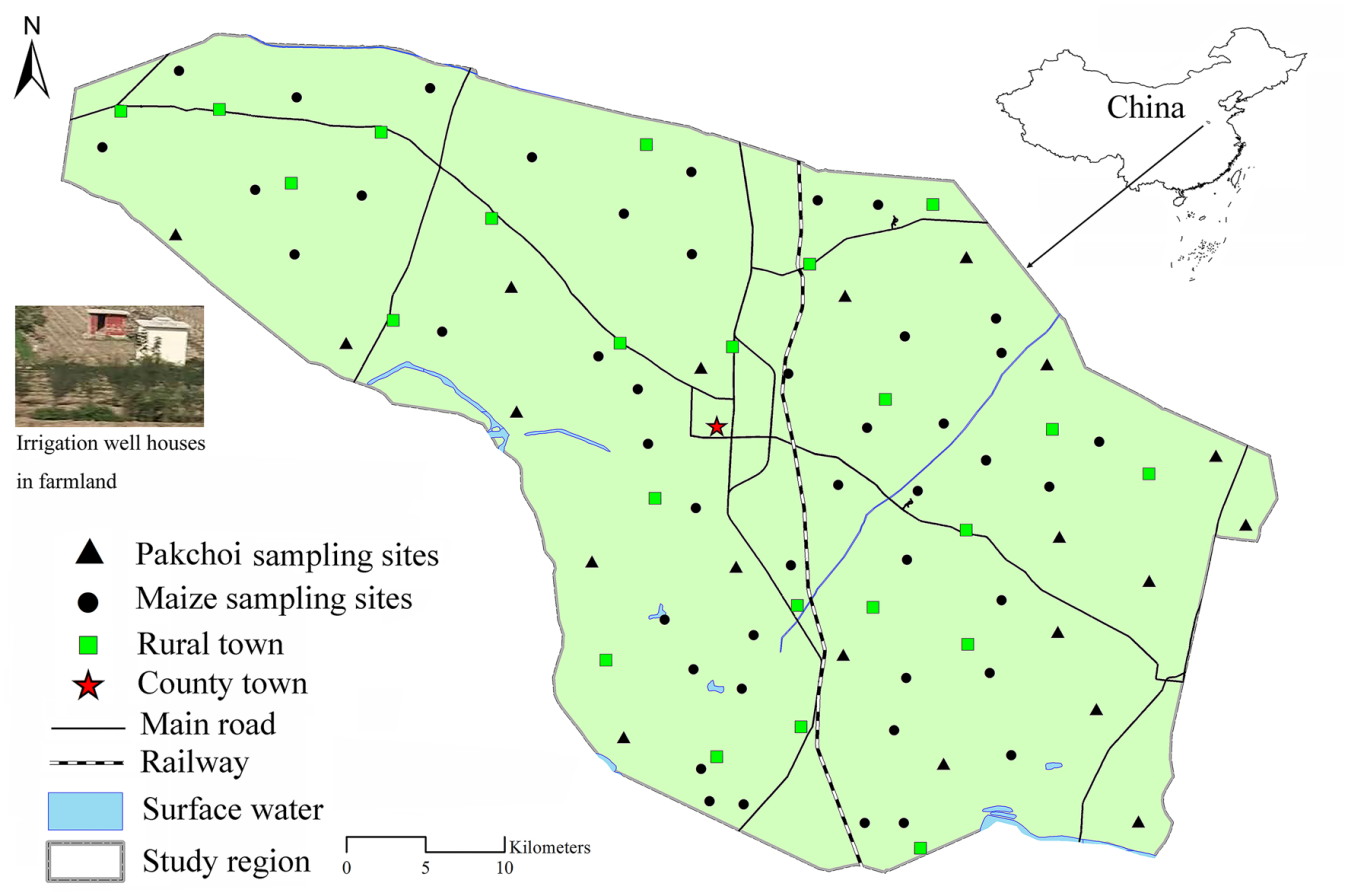
The locations of study area and sampling sites. The boundary was determined according to present situation of land use. The map was obtained from the National Platform for Common Geospatial Information Services (https://www.tianditu.gov.cn/) and created in ArcGIS 10.4 (ESRI, Redlands, CA, USA).

The study area is located in the middle of the North China Plain (Fig. 1). The elevation of the study area rose from 44.8 m to 66.8 m. In 2018, the average temperature and precipitation were 15.4 °C and 697.4 mm, respectively. This area has been the major grain- and vegetable-producing area since ancient times. The main cropping system is double cropping a year, and the area of farmland with triple cropping a year has increased in recent years. The main agricultural soil group is fluvo-aquic soil. Chemical fertilizers, organic fertilizers and pesticides have been widely used in agricultural production since 1980. The chemical fertilizer is mainly N-P-K compound fertilizer, and the organic fertilizer is mainly self-produced from livestock excrement. The pesticides were mainly herbicides and insecticides. The irrigation water is from underground. The main industry is wood processing and clothing, and there are no heavily polluting enterprises in the study area.

### Sample collection

The sampling sites were located in farmland with an irrigation well. The sampling referred to the recommendation methods of Ministry of Agriculture and Rural Affairs of China and Ministry of Ecology and Environment of China^3,28,29^. In October 2018, pakchoi samples were collected from 20 sites, and maize samples were collected from 45 sites (Fig. 1). The soil and irrigation samples were collected with plant sampling. The scope of each sampling area was 200 m × 200 m. According to the stochastic method, nine soil samples (0–20 cm) were collected, and nine entire plant samples were also collected. The nine soil samples and nine plant samples were mixed as one soil sample and one plant sample, respectively. A 2 L glass bottle with a rubber plug was used to collect irrigation water. Before use, the glass bottle and rubber plug were washed with the corresponding irrigation water three times. The pump was turned on to drain water for at least 5 min, and then irrigation water was collected with a glass bottle. The bottle was covered with a rubber plug when it was filled. The irrigation well was in a small house that was 2 m long, 1 m wide and 2 m high. Atmospheric deposition was collected from the roof of the irrigation well house by using a nylon brush. The atmospheric deposition was put in a clean paper bag, and the bag was sealed with scotch tape. Organic and chemical fertilizer and pesticide samples were also collected from farmers and agricultural materials companies in the study area. The collected soil, plant, irrigation water, atmospheric deposition, fertilizer and pesticide samples were placed in a heat retaining box by using an ice bag to maintain 4 °C. The boxes were transported to the laboratory as soon as possible. In the laboratory, the soil samples, deposition samples and fertilizer samples were air-dried and ground, stones and grass debris were removed, and then the samples were stored in sample bottles. The plant samples were washed carefully in deionized water to remove any soil particles and other impurities. The roots and edible parts (grain of maize, leaves of pakchoi) were oven-dried at 105 °C for 40 min and then kept at 75 °C until a constant weight was obtained. The dry plant samples were ground and then dispensed into the sample bottles. All samples were stored in a refrigerator at 4 °C.

### Sample analysis

The soil samples and deposition samples were digested by using an acid digestion mixture (HCl, HNO_3_, HF, and HClO_4_) on an electric hot plate to determine the concentrations of Cd, Pb, Cu and Zn^30^. The DTPA was used to extract the labile fraction of heavy metals from soil^31^. The irrigation water samples were digested with HNO_3_ on an electric hot plate to determine the concentrations of heavy metals^32^. The plant samples were digested with an acid digestion mixture (HNO_3_ and HClO_4_) on an electric hot plate to determine the concentrations of heavy metals^33^. The fertilizer samples were digested with HCl and HNO_3_ on an electric hot plate to determine the concentrations of heavy metals^34^. The pesticide samples were placed in aqua regia in a laboratory microwave system to determine the concentrations of heavy metals^35^. The concentrations of heavy metals in the samples were determined with inductively coupled plasma‒mass spectrometry (ICP‒MS: PerkinElmer NexION 300X; iCAP6300). The standard recovery rates were 93.5–104.2%, and the relative standard deviations (RSD) were less than 5%. This showed high accuracy and precision of the test method. The limit of detection (LOD) and the limit of quantitation (LOQ) were 1.1–12.3 ng L^−1^ and 3.5–38.9 ng L^−1^, respectively.

All methods used to collect and analyze samples were recommended by the Ministry of Agricultural and Rural Affairs and the Ministry of Ecology and Environment of China. Before use, all of the glassware and plastic containers were soaked in 20% (v/v) HNO_3_ for at least 24 h and thoroughly rinsed initially with distilled water and subsequently with deionized water. Furthermore, certified reference samples, comprising bush leaf material (GBW-07603) and yellow soil material (GBW-07408), were used for quality control. The difference between the measured and certified concentrations of elements was no more than 10%.

### Statement of sample collection and analysis

All methods used for collecting and analyzing samples were performed in accordance with the recommendation methods of Ministry of Agriculture and Rural Affairs of China and Ministry of Ecology and Environment of China. The collection of soil, irrigation water and maize samples were permitted by local agricultural department.

### Geostatistical analysis

By using the variation function, the spatial distribution pattern and the correlativity of regionalized variables were investigated in geostatistical analysis^36^. Ordinary kriging interpolation was one of the effective methods. In this study, ordinary kriging interpolation was used to investigate the spatial distribution of the total concentrations and DTPA extraction of heavy metals in soil and the concentrations of heavy metals in roots and edible parts in ArcGIS 10.4 (ESRI, Redlands, CA, USA). The normality test was conducted through Normal QQPlot in ArcGIS 10.4. The test showed that the data obeyed a normal distribution. Cross-validation indicated that the predicted values were close to the measured values.

### Geographical detector method

The geographical detector method could be used to explore the spatially stratified heterogeneity of factors (response factors) and determine the dominant driving factors (explanatory factors)^37,38^. This statistical method was based on the hypothesis that if one independent variable had an important influence on one dependent variable, their spatial distribution was comparable. This method with no linear hypothesis had an elegant form and definite physical meaning. The data of response factors were numeric, and the data of explanatory factors should be discretized as some classifications. The unique advantage of the method was to explore the interaction of two explanatory factors on response factors. By comparing the *q* value of each explanatory factor and the interactive *q* value of two explanatory factors, the existence, intensity, direction and linearity of the interaction could be determined.

The method included four functions: factor detector, interaction detector, ecological detector and risk detector. The factor detector measured the influence of explanatory factors on the response factor. A higher *q* value indicated a higher influence. The interaction detector revealed whether two explanatory factors had an interactive influence on the response factor. The interaction types are shown in Table 1. The ecological detector identified the difference in the impacts of two explanatory factors, which was assessed by the *F* statistic. The risk detector indicated the significance of the difference between the average values of the response factor in each stratum of the explanatory factor. The relevant calculations were conducted through GeoDetector software based on Microsoft Excel (http://geodetector.cn/).

**Table 1:** Interaction between explanatory variables (*X1*, *X2*).

Description	Interaction
*q*(*X1* ∩ *X2*) < Min (*q*(*X1*), *q*(*X2*))	Weaken, nonlinear
Min(*q*(*X1*), *q*(*X2*)) < *q*(*X1* ∩ *X2*) < Max(*q*(*X1*), *q*(*X2*))	Weaken, univariate
*q*(*X1* ∩ *X2*) > Max(*q*(*X1*), *q*(*X2*))	Enhance, bivariate
*q*(*X1* ∩ *X2*) = *q*(*X1*) + *q*(*X2*)	Independent
*q*(*X1* ∩ *X2*) > *q*(*X1*) + *q*(*X2*)	Enhance, nonlinear

### Establishment of the factor system and data sources

The factors system included response factors and explanatory factors. The main purpose of this research was to investigate the effects of explanatory factors on response factors and to determine the dominant explanatory factors. The four response factors included the total concentration and DTPA extraction of Cd, Pb, Cu and Zn in soil and the concentrations of heavy metals in root and edible parts. Eleven explanatory factors are shown in Table 2. Soil type (ST) concerns the natural sources of heavy metals. In addition, ST was the property deciding the capacity of soil fertility maintenance and supply and therefore affected agricultural practice^39^. Soil fertility grade (SFG) was the main basis for the application of STFF, influencing the use of fertilizer and pesticides. Irrigation frequency (IF) and heavy metal concentration in irrigation water (HMCIW) were selected as irrigation factors that were considered to affect heavy metal input in agricultural soil^18^. The management of reducing the use of fertilizer and pesticides (MRUFP) was selected as the policy factor, including the implementary measures of STFF and pesticide reduction. The STFF and APPR projects were conducted in the study area in 2007 and 2015, respectively. In the study area, the MRUFP included standard and nonstandard values. The standard MRUFP referred to the application of fertilizer and pesticides strictly following the recommendation of STFF and APPR, and excess application of fertilizer and pesticides occurred under the nonstandard MRUFP. Applying the quantity of organic fertilizer (AQOF), applying the quantity of chemical fertilizer (AQCF) and applying the quantity of straw (AQS) were selected as fertilization factors that have been demonstrated to affect heavy metal concentrations in farmland^21,40^. In addition, the applied quantity of herbicide (AQH) and applied quantity of insecticide (AQI) were selected as pesticide factors. The major source of heavy metals in agricultural soils of the North China Plain was demonstrated to be atmospheric deposition in recent years. The heavy metal concentration in atmospheric deposition (HMCAD) was selected as the explanatory factor. In summary, explanatory factors included six types: soil factors (ST, SFG), irrigation factors (IF, HMCIW), policy factors (MRUFP), fertilization factors (AQOF, AQCF, AQS), pesticides factors (AQH, AQI) and atmospheric deposition factor (HMCAD).

**Table 2:** The types and discretization of explanatory factors.

Types of explanatory factors	Explanatory factors	Levels (contents)	Discretization methods
Soil factors	Soil type (ST)^①^	1 (Sulfate saline fluvo-aquic soil), 2 (Chloride saline fluvo-aquic soil), 3 (Loamy fluvo-aquic soil), 4 (Loamy browning fluvo-aquic soil), 5 (Clay browning fluvo-aquic soil), 6 (Sandy fluvo-aquic soil), 7 (Soda chloride saline fluvo-aquic soil), 8 (Clay fluvo-aquic soil)	Discretized according to name
	Soil fertility grade (SFG)^②^	1 (grade 1), 2 (grade 2), 3 (grade 3), 4 (grade 4), 5 (grade 5), 6 (grade 6)	Discretized according to classification
Irrigation factors	Irrigation frequency (IF)^②^	1 (Irrigation once a year), 2 (Irrigation twice a year), 3 (Irrigation three times a year)	Discretized according to classification
	Heavy metal concentration in irrigation water (HMCIW, μg L^−1^)^②^	Cd: 1 (0–0.03), 2 (0.03–0.06), 3 (0.06–0.1) Pb: 1 (0–0.01), 2 (0.01–0.02), 3 (0.02–0.03) Cu: 1 (0–0.3), 2 (0.3–0.6), 3 (0.6–0.9) Zn: 1 (0–0.6), 2 (0.6–1.2), 3 (1.2–1.8)	Equal interval method
Policy factor	Management of reducing the use of fertilizer and pesticides (MRUFP)^②^	1 (Standard), 2 (Nonstandard)	Discretized according to classification
Fertilization factors	Applying quantity of organic fertilizer (AQOF, t hm^−2^ a^−1^)^③^	Maize: 1 (3–7.5), 2 (7.5–12), 3 (12–16.5), 4 (16.5–21) Pakchoi: 1 (6–10.5), 2 (10.5–15), 3 (15–19.5), 4 (19.5–24)	Equal interval method
	Applying quantity of chemical fertilizer (AQCF, kg hm^−2^ a^−1^)^③^	Maize: 1 (100–325), 2 (325–550), 3 (550–775), 4 (775–1000) Pakchoi: 1 (150–450), 2 (450–700), 3 (700–1050), 4 (1050–1350)	Equal interval method
	Applying quantity of straw (AQS, t hm^−2^ a^−1^)^②^	1 (3–4), 2 (4–5), 3 (5–6)	Equal interval method
Pesticides factors	Applying quantity of herbicide (AQH, kg(L) hm^−2^ a^−1^)^③^	Maize: 1 (1.5–1.95), 2 (1.95–2.4), 3 (2.4–2.85) Pakchoi: 1 (1.8–2.25), 2 (2.25–2.7), 3 (2.7–3.15)	Equal interval method
	Applying quantity of insecticide (AQI, kg(L) hm^−2^ a^−1^)^③^	Maize: 1 (2.1–2.55), 2 (2.55–3), 3 (3–3.45) Pakchoi: 1 (2.4–2.85), 2 (2.85–3.3), 3 (3.3–3.75)	Equal interval method
Atmospheric deposition factor	Heavy metal concentration in atmospheric deposition (HMCAD, mg kg^−1^)^②^	Cd: 1 (0.4–0.45), 2 (0.45–0.5), 3 (0.5–0.55), 4 (0.55–0.6) Pb: 1 (30–35), 2 (35–40), 3 (40–45), 4 (45–50) Cu: 1 (60–65), 2 (65–70), 3 (70–75), 4 (75–80) Zn: 1 (140–145), 2 (145–150), 3 (150–155), 4 (155–160)	Equal interval method

Note: ①: the levels of explanatory factors for pakchoi were divided from 1 to 5, and the levels of explanatory factors for maize were divided from 1 to 8; ②: the levels were suitable for explanatory factors of both maize and pakchoi; ③: different levels were suitable for maize or pakchoi.

The data of all response factors and some explanatory factors (HMCIW, HMCAD) were obtained through laboratory analysis. The data for ST, SFG, IF, ASTFF, AQOF, AQCF, AQH, AQI, and AQS were provided by the local agricultural administration department and were verified through our survey of local farmers. The discretization of explanatory factors was conducted by using the methods in a previous study^41^.

## Results

### Discretization of explanatory factors

According to the discretization method, each explanatory factor was classified into different levels (Table 2). The results of the risk detector showed that the average values of the response factor at different levels of each explanatory factor were significantly different (*p* < 0.05; detailed data are not entirely shown for a large amount of data). This indicated that the selected discretization method was optimal. The great soil group of collected soil samples was fluvo-aquic soil, including eight soil genera. Nearly two-thirds of the soil samples were loamy fluvo-aquic soil, which had high arability. The soil fertility of more than 60% of the soil samples was moderate (soil fertility grades 2, 3 and 4), and only three soil samples were high (soil fertility grade 1). The proportions of each soil fertility grade between maize soil and pakchoi soil were almost the same. Over half of the sampling sites of maize or pakchoi were irrigated more than once a year, and heavy metal concentrations in nearly half of the irrigation water samples for maize or pakchoi belonged to a high level (level 3). The concentrations of Cd, Pb, Cu and Zn were far less than the limitation of heavy metals in irrigation water (Cd: 10 μg L^−1^, Pb: 200 μg L^−1^, Cu: 1000 μg L^−1^, Zn: 2000 μg L^−1^) in China^29^. More than 70% of maize or pakchoi sampling sites were under standard MRUFP, with low application quantity of fertilizers (Levels 1, 2 and 3) and pesticides (Levels 1 and 2). In those sampling sites under nonstandard MRUFP (less than 30%), excess fertilizers and pesticides were applied to ensure the production of crops and vegetables. Similarly, the HMCAD of most sampling sites with standard MRUFP belonged to the low level (levels 1, 2 and 3), and the HMCAD of sampling sites with nonstandard MRUFP belonged to level 4. The sampling sites of each level of AQS were almost the same.

### Spatial distribution of heavy metals in soil and plants

**Figure 2:**
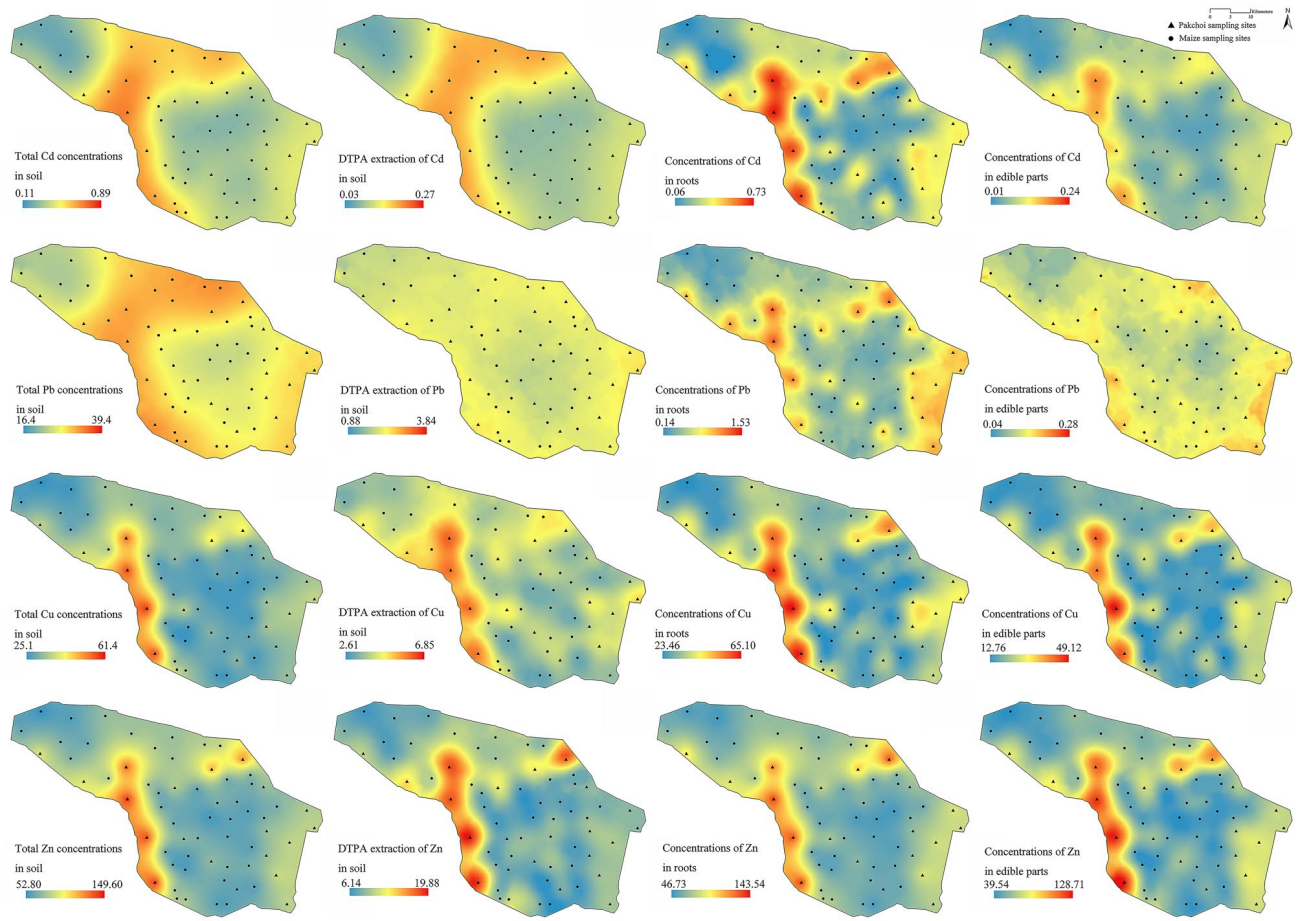
The spatial distribution of Cd, Pb, Cu and Zn in soil and plant. The unit was mg kg^−1^.

The concentrations of Cd and Pb in soil and plants were higher in the northwest-central part of the study region than in other parts (Fig. 2). The concentrations of Cu and Zn in soil and plants were higher in the western and northern parts of the study region than in other parts. The average values of heavy metals in soil and plants were higher in pakchoi sampling sites than in maize sampling sites (Table 3). Except for the total concentrations of Pb in 23 sampling sites of maize and the total concentrations of Zn in 20 sampling sites of maize, the total concentrations of heavy metals in the soil of other sampling sites were more than the background values in regional soil (Table 3). This indicated the accumulation of heavy metals in the farmland soil of the study region. Except for total concentrations of Cd in 4 sampling sites of maize and 6 sampling sites of pakchoi, total concentrations of heavy metals in soil of other sampling sites were less than the risk screening values for soil contamination of agricultural land in China (Table 3). Except for concentrations of Cd in edible parts of 3 maize sampling sites and 3 pakchoi sampling sites, concentrations of heavy metals in edible parts of other sampling sites were less than the limitations of food in China (Table 3). This indicated light pollution of Cd in agricultural soils of the study area.

**Table 3:** Average value of response factors, background value of heavy metals in regional soil and limitation of heavy metals in soil and food (mean ± SD, mg kg^−1^).

Sampling sites	Average value	Cd	Pb	Cu	Zn
Maize	Total concentration in soil	0.342 ± 0.177	25.794 ± 5.631	29.118 ± 3.515	66.633 ± 8.196
	DTPA extraction in soil	0.103 ± 0.054	1.847 ± 0.561	3.693 ± 0.616	8.293 ± 1.268
	Concentration in roots	0.189 ± 0.098	0.273 ± 0.079	28.982 ± 3.852	59.782 ± 8.289
	Concentration in edible parts	0.050 ± 0.029	0.088 ± 0.027	15.938 ± 2.266	50.232 ± 6.801
Pakchoi	Total concentration in soil	0.555 ± 0.157	33.965 ± 3.379	41.145 ± 10.172	98.87 ± 28.656
	DTPA extraction in soil	0.163 ± 0.053	2.847 ± 0.502	4.967 ± 0.999	12.087 ± 4.258
	Concentration in roots	0.478 ± 0.128	1.217 ± 0.167	45.249 ± 10.536	96.06 ± 28.593
	Concentration in edible parts	0.135 ± 0.043	0.218 ± 0.032	30.973 ± 7.857	83.682 ± 23.674
Background value in regional soil^42^		0.084	25.8	24.0	63.5
Risk screening values for heavy metals of farmland in China^3^		0.6	170	100	300
Limitation of heavy metals for food in China^43^	Maize	0.1	0.2	–	–
	Pakchoi	0.2	0.3	–	–

### Influence of explanatory factors on heavy metals in soil and plants

**Figure 3:**
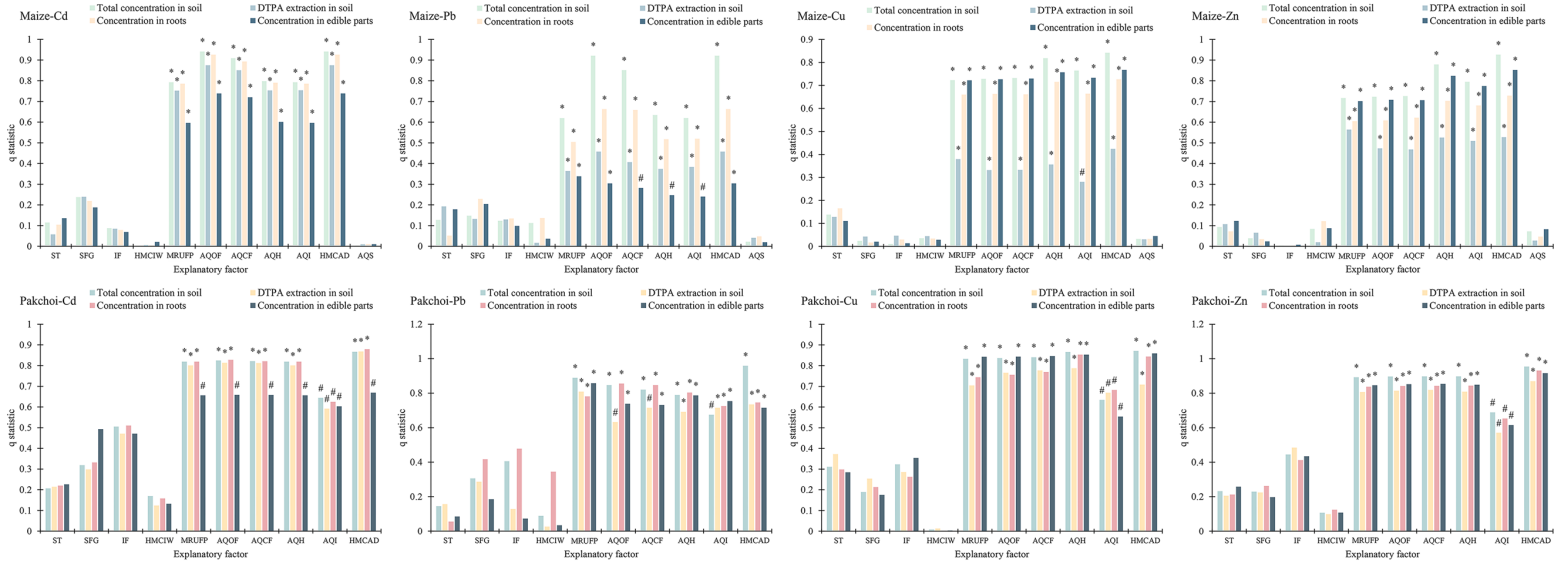
The results of factor detector. *: *q* statistic is significant at the 0.01 level; #: *q* statistic is significant at the 0.05 level.

**Figure 4:**
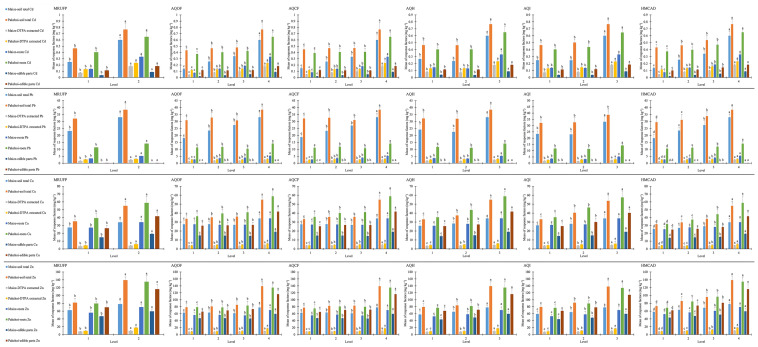
The partial results of risk detector. Each column showed average value of related response factor of the sampling sites on corresponding level of explanatory factor. Different lowercase letters on the columns indicate significant differences at p < 0.05 based on the one-way analysis of variance (ANOVA).

**Figure 5:**
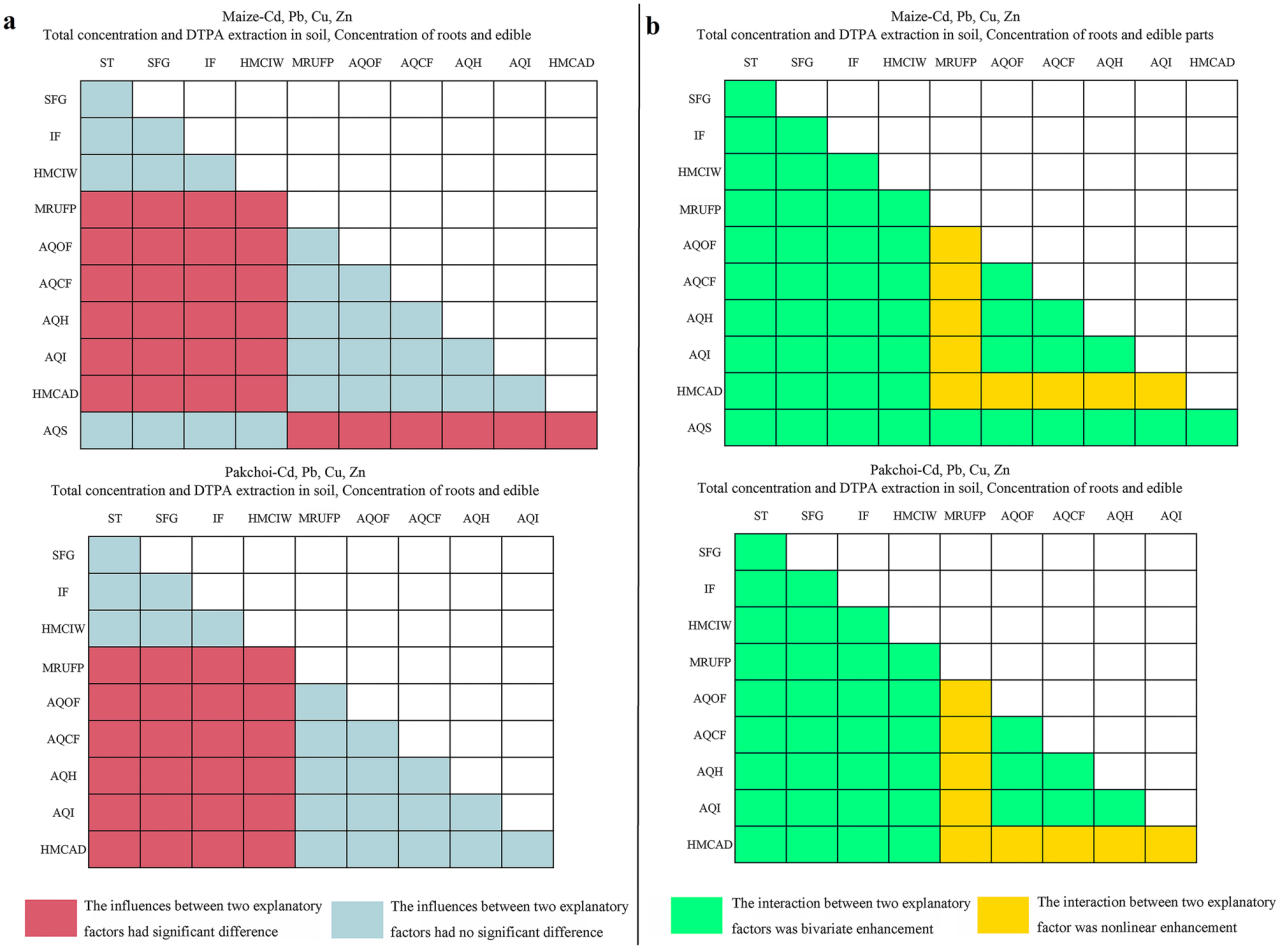
The results of ecological detector (**a**) and interaction detector (**b**).

The results of the factor detector showed a significant influence of MRUFP, AQOF, AQCF, AQH, AQI and HMCAD on heavy metals in soil and plants (Fig. 3). The results of the risk detector showed that the response factors increased significantly with increasing levels of these explanatory factors (Fig. 4, *p* < 0.05). The higher concentrations of heavy metals in soil and plants included the sampling sites with nonstandard MRUFP, high HMCAD and high application amounts of fertilizers and pesticides, and vice versa. The results of the ecological detector showed that the influences of MRUFP, AQOF, AQCF, AQH, AQI, and HMCAD had significant differences from those of other explanatory factors on the heavy metal concentrations of soil and plants in maize or pakchoi sampling sites, and the influences were not significantly different among MRUFP, AQOF, AQCF, AQH, AQI, and HMCAD (Fig. 5, *p* < 0.05). This indicated that these explanatory factors did have significant influences on response factors. The results of the interaction detector showed that the interactions between MRUFP and AQOF, AQCF, AQH, and AQI and between HMCAD and MRUFP, AQOF, AQCF, AQH, and AQI were nonlinearly enhanced, and the interactions between other explanatory factors were bivariate enhanced (Fig. 5, *p* < 0.05). This indicated that MRUFP greatly enhanced the influences of AQOF, AQCF, AQH, and AQI on the heavy metal concentrations of soil and plants at both the maize and pakchoi sampling sites, and the influence of HMCAD was greatly enhanced by MRUFP, AQOF, AQCF, AQH, and AQI.

## Discussions

Previous studies in South China showed that irrigation water was the main source of heavy metals in agricultural soils^18,44^. However, we found that the influences of irrigation factors (IF and HMCIW) on heavy metals in soil and plants were not significant. This might be attributed to the low concentrations of heavy metals in irrigation water and limited irrigation frequency in this study. Until now, the driving effects of straw return on the accumulation and bioavailability of heavy metals in agricultural soil have remained controversial^17,45^. The decomposition of straw in soil is a complex process, inducing different interactions between the decomposition products and heavy metals. We found that the effect of straw return on heavy metal accumulation in soil and plants was not significant. This might be attributed to the practices of agricultural production. In the study area, straw burning was prohibited. Waste straw was preferentially sold to livestock and poultry farms as fodder, and the rest was applied to farmland. Since 2015, reasonably increasing the application of straw has been encouraged to increase organic matter in agricultural soils and reduce the application amounts of chemical fertilizer. The straw return was not completely based on the demand of soil fertility and was highly random. Furthermore, the heavy metal concentration in straw was low. These factors induced the inapparent influence of straw return.

Atmospheric deposition, rather than fertilization and pesticide application, was suggested to be the dominant element source of heavy metals in agricultural soil, and the heavy metals in atmospheric deposition mainly came from heavy industry and coal combustion^21^. However, the accumulation of Cd, Pb, Cu and Zn in farmland in the North China Plain was demonstrated to be mainly due to long-term agricultural practices, such as fertilizer and pesticide application, and atmospheric deposition was the secondary exogenous source of heavy metals^46^. Another study conducted on the North China Plain indicated that the accumulation of heavy metals in soil was related to the application of organic fertilizer, phosphate fertilizer and compound fertilizer^15^. In this study, the results of the Geographical Detector also indicated the dominant driving effects of atmospheric deposition and the application of fertilizers and pesticides on the accumulation of heavy metals in soil and plants. In North China, airborne heavy metals are abundant in coarse particles and mainly settle in the regions around pollution sources^47^. Therefore, some heavy metal pollution sources were distributed around the agricultural soil with the accumulation of related heavy metals. Through the survey, there were no other heavy metal pollution sources in or around the study region. The soot emissions from the involved industries were sharply reduced due to the implementation of regional air pollution prevention and control plans from 2013 to 2018. By the end of 2017, coal-fired boilers with or less than 100 kiloton vapors were dismantled, and the pollutant emissions of coal-fired boilers with more than 100 kiloton vapors were strictly controlled. The heavy metals in atmospheric deposition might come from the dust raised by wind from surrounding farmland. Moreover, the results of the interaction detector showed that the management of reducing the use of fertilizers and pesticides and the application amounts of fertilizers and pesticides greatly enhanced the influence of atmospheric deposition on the heavy metal concentrations of soil and plants. Thus, the influences of atmospheric deposition and other significant explanatory factors on the accumulation of heavy metals in soil and plants were equally important.

In the study area, heavy metals were found in organic and chemical fertilizers (Table 4). The concentrations of heavy metals in organic fertilizers were far more than those in chemical fertilizers. The application amounts of organic fertilizers in all sampling sites were more than the average values (2.25 t hm^−2^) of the country^48^. The application amounts of organic fertilizers in most sampling sites were more than the average values of the country (369.58 kg hm^−2^, data from Food and Agriculture Organization of the United Nations, https://www.fao.org/faostat). The application of organic and chemical fertilizers induced the accumulation of heavy metals in farmland. The application of pesticides could induce heavy metal accumulation in agricultural soils^49,50^. Different concentrations of Cd, Pb, Cu and Zn were detected in the collected pesticides (herbicide and insecticide, Table 4). In addition, the application amounts of herbicides and insecticides were far more than those of pesticides (total) for China in 2018 (2.17 kg(L) hm^−2^, data from Food and Agriculture Organization of the United Nations, https://www.fao.org/faostat). The application of herbicides and insecticides would increase heavy metals in farmland. In brief, atmospheric deposition and the excess application of fertilizer and pesticides and atmospheric deposition directly caused the accumulation of Cd, Pb, Cu and Zn in agricultural soils in the study area.

**Table 4:** Concentrations of heavy metals in fertilizers and pesticides.

	Organic fertilizers	Chemical fertilizers	Herbicide	Insecticide
Number of samples	40	40	28	26
Concentration range (mg kg^−1^)				
Cd	0.53–5.87	0.10–0.96	0.003–0.86	0.008–1.27
Pb	9.74–49.62	0.91–1.57	0.42–1.68	1.05–3.54
Cu	36.94–341.28	5.27–13.43	6.41–10.58	11.32–19.75
Zn	113.86–664.27	12.85–38.45	16.78–64.86	20.67–84.21
Limitation^51^ (mg kg^−1^)				
Cd	3	10	–	–
Pb	50	200	–	–
Cu	–	–	–	–
Zn	–	–	–	–

The factor detector indicated the significant influences of the management policy of reducing the use of fertilizers and pesticides and atmospheric deposition on the accumulation of Cd, Pb, Cu and Zn in soil and plants. Moreover, the interaction detector indicated the greatly enhanced influences of fertilization, pesticide use and atmospheric deposition by MRUFP. Of the four types of driving factors, MRUFP was the dominant factor. Actually, the high application level of fertilizers and pesticides and the high concentration level of heavy metals in atmospheric deposition were distributed in the region with nonstandard MRUFP. The region with nonstandard application of MRUFP included approximately half of the high and low soil fertility levels of farmland in the study area. In farmland with high soil fertility, the area of farmland with triple cropping a year increased by year. High-intensity agricultural production activities require the abundant application of fertilizers and pesticides to ensure the yield and quality of agricultural products. In farmland with low soil fertility, huge fertilizers were applied to improve soil fertility, and abundant pesticides were also used to ensure the yield and quality of agricultural products. Despite the application of STFF and the reduced use of pesticides in the whole study area, the management has gradually loosened since 2011. In these regions, the application of fertilizers and pesticides has increased since 2011 due to the needs of agricultural production, inducing the accumulation of heavy metals in soil and plants. As no other heavy metal pollution sources were distributed in or around the study area, heavy metals in atmospheric deposition in dust were raised by wind from the surrounding farmland. This induced atmospheric deposition with high concentrations of heavy metals was to be distributed in regions with nonstandard MRUFP, and vice versa. In view of this, reducing the use of fertilizers and pesticides requires strict management measures to prevent heavy metal accumulation in agricultural soils and products.

Through the analysis of heavy metal concentrations in collected organic and chemical fertilizer samples, it was found that the concentrations of heavy metals in organic fertilizers were far more than those in chemical fertilizers, and the concentrations of Cd and Pb in some organic fertilizer samples exceeded the limitations of fertilizers in China (Table 4). This was in accordance with the results of content analysis of heavy metals in common fertilizers in typical north vegetable fields of China^44^ and in conflict with the results of foreign research^50^. Foreign research has shown that the concentrations of Cd, Pb, Cu and Zn in chemical fertilizers are far more than those in manure fertilizers. This was attributed to the strict quality control of chemical fertilizer in China. In the study area, most organic fertilizers were self-produced from livestock manure bought from livestock and poultry farms by farmers, and commercial organic fertilizers were not widely used due to their high price. The quality of self-produced organic fertilizers could not be guaranteed. In addition, since 2015, increasing organic fertilizers have been suggested to substitute for the application of chemical fertilizers. Thus, the application amounts of organic fertilizers in the study region were far more than those of chemical fertilizers (Table 2). Therefore, organic fertilizers contributed more heavy metals to agricultural soil than chemical fertilizers. A previous study also showed that substituting chemical fertilizer with organic fertilizer induced the accumulation of Cd, Pb, Cu and Zn in soil^52^. To prevent the accumulation of heavy metals in agricultural soils and products, the application of organic fertilizers needed to follow the recommendations of STFF. Under the premise of price control, substituting self-produced organic fertilizers with standardized commercial organic fertilizers was an effective method. Normative monitoring of heavy metals in commercial organic fertilizers is essential to guarantee quality.

## Conclusion

In this study, a major grain- and vegetable-producing area with accumulation of Cd, Pb, Cu and Zn in the soil of the North China Plain was selected as the study area. The geographical detector method was used to determine the driving factors of the accumulation of Cd, Pb, Cu and Zn in agricultural soils and products. Policy factors (management of reducing the use of fertilizer and pesticides), fertilization factors (application of organic and chemical fertilizers), pesticide factors (application of herbicides and insecticides) and atmospheric deposition factors (heavy metal concentration in atmospheric deposition) had significant influences on the accumulation of heavy metals in soil and plants. Among these factors, the policy factor was the dominant driving factor that greatly enhanced the influences of the other three types of factors. Atmospheric deposition and the excess application of fertilizers and pesticides directly induce the accumulation of heavy metals in soil and plants. Organic fertilizers contribute high levels of heavy metals to agricultural soil because of their high concentrations of heavy metals and abundant application amounts. The application of formulated fertilization and action plans for pesticide reduction effectively decreased the accumulation of heavy metals in soil and plants. The standard application of organic fertilizers and normative monitoring of heavy metals in organic fertilizers were suggested to prevent heavy metal accumulation in agricultural soils of the study area.

## Data Availability

The datasets used and/or analyzed during the current study are available from the corresponding author on reasonable request.
